# The impact of symptom duration on D-dimer: results from the Venous Thrombosis Registry in Østfold Hospital

**DOI:** 10.1016/j.rpth.2026.103457

**Published:** 2026-03-26

**Authors:** Mazdak Tavoly, Camilla Tøvik Jørgensen, Katarina Glise Sandblad, Waleed Ghanima, Lamya Garabet

**Affiliations:** 1 Department of Medicine, Geriatrics and Emergency Medicine, Sahlgrenska University Hospital/Sahlgrenska, Region Västra Götaland, Gothenburg, Sweden; 2 Department of Research, Østfold Hospital, Sarpsborg, Norway; 3 Department of Emergency Medicine, Østfold Hospital, Norway; 4 Department of Molecular and Clinical Medicine, Institute of Medicine, Sahlgrenska Academy, University of Gothenburg, Gothenburg, Sweden; 5 Region Västra Götaland, Sahlgrenska University Hospital/Östra, Department of Medicine, Geriatrics and Emergency Medicine, Gothenburg, Sweden; 6 Institute of Clinical Medicine, University of Oslo, Østfold, Norway; 7 Center for Laboratory Medicine, Østfold Hospital Trust, Grålum, Norway; 8 Multidisciplinary Laboratory Medicine and Medical Biochemistry, Akershus University Hospital, Lørenskog, Norway

**Keywords:** clinical laboratory services, fibrinogen, pulmonary embolism, registries, venous thromboembolism

## Abstract

**Background:**

It has been suggested that D-dimer testing should be avoided in patients with prolonged symptoms, as results may yield false negatives. However, the evidence supporting this recommendation is limited.

**Objectives:**

This study evaluated the impact of symptom duration on D-dimer and investigated associations between false-negative D-dimer results and prolonged symptoms.

**Methods:**

Patients diagnosed with pulmonary embolism, deep vein thrombosis (DVT), or upper extremity DVT between 2005 and 2020 were identified through the Venous Thrombosis Registry in *Ø*stfold Hospital (TROLL), Norway. Eligible patients had available D-dimer results and symptom duration data. Correlations between symptom duration and D-dimer were evaluated using Kendall’s τ test. The association between false-negative D-dimer results and symptom duration categories (0-7 days, 8-14 days, and beyond 15 days) was analyzed using the Cochrane–Armitage test.

**Results:**

In total 3423 patients were included, of whom 1696 (49.5%) were women, with a median age of 67 years (IQR, 54-78 years). The median symptom duration was 4 days (IQR, 2-10 days), and 946 patients (27.6%) reported symptoms exceeding 1 week. D-dimer showed a weak negative correlation with symptom duration (Kendall τ coefficient = −0.04; *P* < .001). Overall, 20 patients (0.6%) had false-negative D-dimer results, but these were not significantly associated with symptom duration (*P* = .55).

**Conclusion:**

Although D-dimer were negatively correlated with symptom duration, the coefficient was very weak and unlikely to have clinical relevance. Moreover, false-negative D-dimer results were not associated with prolonged symptoms, suggesting that extended symptom duration may not affect the performance of D-dimer testing in clinical practice.

## 1 Introduction

Available guidelines for the acute diagnostic management of venous thromboembolism (VTE) recommend that D-dimer testing should only be performed in patients with low or intermediate pretest probability [1,2]. However, this recommendation has been challenged by recent studies, demonstrating that a D-dimer <0.5 mg/L fibrinogen equivalent units can reliably exclude acute VTE with acceptable failure rates, regardless of clinical probability [3,4]. In addition, these studies suggest that broader D-dimer testing increases diagnostic efficiency by reducing referrals for imaging, thereby shortening visit times and lowering health care costs [5]. Nevertheless, concerns remain regarding the reliability of D-dimer testing in patients with a high clinical probability of VTE. Similarly, symptom duration has also been argued to influence D-dimer, with prolonged symptoms may potentially lead to falsely negative results [6,7]. In this context, D-dimer testing in patients presenting with symptoms persisting for >1 to 2 weeks has been discouraged [8,9]. However, this argument is largely based on studies from the 1990s [6,7] and a small number of retrospective studies with limited sample sizes [10,11]. A post hoc analysis combining data from 2 large prospective outcome studies suggested that symptom duration does not increase the risk of false-negative D-dimer results [12]. However, this study included only patients with pulmonary embolism (PE) and was conducted before the introduction of age-adjusted D-dimer thresholds. Importantly, none of the available prospective diagnostic management studies of VTE have excluded patients with prolonged symptom duration [4,13,14]. Although the median duration of symptoms in these studies was <7 days, it is reasonable to assume that some patients with longer symptom durations (>1-2 weeks) were included. Still, no reports from these studies are available that suggest an association between the false-negative D-dimer results and prolonged symptoms. Despite this, many physicians remain reluctant to use D-dimer testing in patients with prolonged symptoms.

We therefore used the Venous Thrombosis Registry in Østfold Hospital (TROLL) to identify patients with available data on both D-dimer results and symptom duration. The objectives of this study were to (i) evaluate the impact of symptom duration on D-dimer, (ii) characterize patients with false-negative D-dimer and age-adjusted D-dimer results, and (iii) assess whether false-negative D-dimer results were associated with prolonged symptoms.

## 2 Methods

### 2.1 Study design and setting

This was a study based on data retrieved from the TROLL registry, which is an ongoing, prospective, single-center, quality control, and research registry of unselected and consecutive patients with VTE who were diagnosed, treated, and followed up at Østfold Hospital, Norway, since 2005 [15]. Serving as the primary referral center for Østfold county, the hospital covers a population of 317,000 inhabitants. The details of the TROLL registry have been previously published [15]. Briefly, all patients who are diagnosed with VTE and/or treated for VTE at Østfold Hospital are referred to the outpatient clinic at the hospital, where they are followed up.

The study was approved by the Regional Committee for Medical and Health Research Ethics (reference number 267223). Participants who were alive and had provided an informed written consent for research participation were included, as well as deceased patients, for whom consent was waived by the Regional Committee for Medical and Health Research Ethics.

### 2.2 Inclusion and exclusion criteria

All patients aged ≥18 years with a VTE diagnosis between 2005 and 2020 were eligible for study inclusion. The inclusion criteria consisted of (a) VTE confirmed by compression ultrasound or phlebography in cases of deep vein thrombosis (DVT) in the lower extremities or upper extremity DVT, computed tomography pulmonary angiography, ventilation-perfusion scintigraphy, or pulmonary angiography in cases of PE, or autopsy-verified VTE as the final cause of death; (b) available D-dimer measurement; and (c) recorded symptom duration. All cases with symptom duration >3 months were checked against the medical records an additional time to ensure the correctness of the registered data. Exclusion criteria consisted of VTE diagnosed in locations other than DVT, PE, and upper extremity DVT.

### 2.3 Variables

Recorded patient characteristics included age; sex; body mass index (in kg/m^2^); a history of VTE; provoked and unprovoked VTE event based on the presence of risk factors such as surgery, trauma, or immobilization within the preceding 12 weeks; use of estrogen-containing contraceptives; hormone replacement therapy; pregnancy or puerperium; long-haul flights (>4 hours) within the last 12 weeks; and active cancer, defined as cancer diagnosed within the preceding 6 months or ongoing anticancer treatment.

### 2.4 D-dimer assay

During the entire study period the STA-Liatest D-Di assay (Diagnostica Stago) was used. In 2016, the assay was upgraded to the STA-Liatest D-Di plus assay, which was developed to reduce analytical interference and improve specificity. At all times, a D-dimer threshold of <0.5 μg/mL fibrinogen equivalent units was regarded as a negative result.

### 2.5 Study outcomes

The primary outcome was the median D-dimer across 3 predefined symptom duration intervals: 0 to 7 days, 8 to 14 days, and ≥15 days. These intervals were chosen based on both clinical and methodological considerations. Clinically, prior literature suggests a threshold of 1 to 2 weeks as the point beyond which D-dimer testing may be less reliable [8,9]. Methodologically, categorization was required to account for the expected skewed distribution of symptom duration. In addition, the correlation between D-dimer and symptom duration was evaluated. The secondary outcome was the proportion of false-negative D-dimer results, assessed using both the conventional threshold of 0.5 μg/mL and the age-adjusted threshold (age × 0.01 for patients > 50 years) [16], stratified by the same symptom duration intervals.

### 2.6 Statistical analyses

Continuous variables are presented as medians with corresponding IQRs. Normality of D-dimer and symptom duration was checked using the Shapiro–Wilks test, which demonstrated substantial skewness for both variables. Median D-dimer across the predefined symptom duration intervals were compared using the Kruskal–Wallis test. Scatter plots with locally estimated scatterplot smoothing were used to visually explore trends in D-dimer according to symptom duration. The association between D-dimer and symptom duration was assessed using Kendall τ rank correlation test, with 95% CI reported for the τ coefficient. This test was chosen over Spearman ρ due to the large number of tied values in the symptom duration variable [17]. Potential associations between false-negative D-dimer rates—using both conventional and age-adjusted thresholds—and symptom duration were examined with the Cochran–Armitage test for trend.

Because not all patients in the TROLL registry were subjected to D-dimer testing and information regarding clinical pretest probability was unavailable, a sensitivity analysis was performed including only patients with DVT diagnosed between 2017 and 2020. This approach was based on the Ri-schedule study, a diagnostic management study conducted at Østfold Hospital, which evaluated the safety of D-dimer testing as a standalone strategy in patients with suspected DVT [3]. The study demonstrated acceptable failure rates for standalone D-dimer testing, leading to its implementation in standard care. Accordingly, we hypothesized that restricting analyses to this subgroup would allow assessment of all patients with DVT, rather than only those with available D-dimer results.

All analyses were performed using the statistical software package R version 4.4.3 (R Foundation for Statistical Computing). Statistical significance was set at an α of 0.05, and all *P* values were 2-sided.

## 3 Results

**Figure 1:**
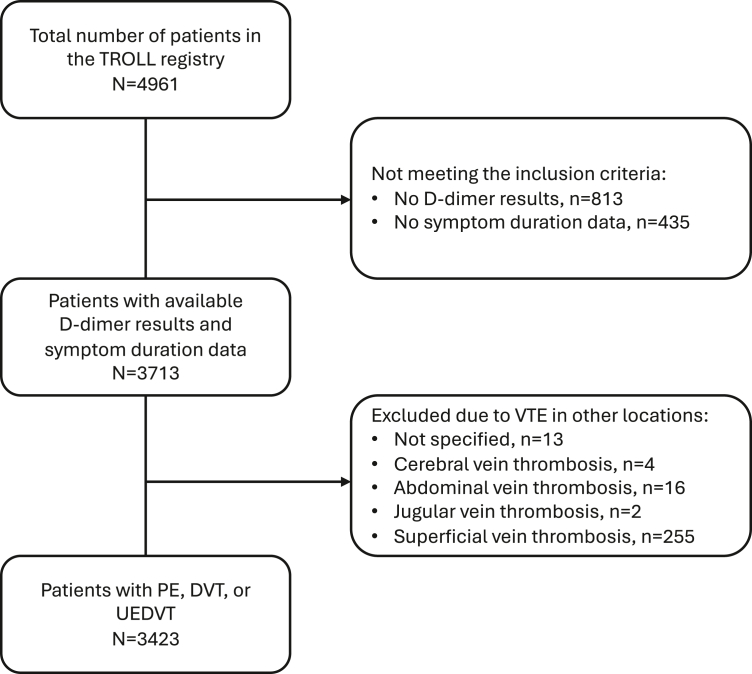
Study flow chart. DVT, deep vein thrombosis; PE, pulmonary embolism; UEDVT, upper extremity DVT; VTE, venous thromboembolism.

The study flow chart is displayed in Figure 1. By May 2020, 4961 patients had been recorded in the TROLL registry, of whom 4148 (83.6%) patients had been subjected to D-dimer testing. Of these, 435 (10.5%) patients lacked information regarding either symptom duration or were assessed as asymptomatic. An additional 255 (6.1%) were diagnosed with only superficial thrombosis and were excluded. The final study cohort consisted of 3423 (82.5%) patients (Figure 1). Baseline characteristics of patients excluded due to unavailable D-dimer results or symptom duration data are presented in Supplementary Table 1.

Patient characteristics are displayed in Table 1. The median age of the overall cohort was 67 years (IQR, 54-78 years), and 1612 (47.0%) were women. Median symptom duration was 4 days (IQR, 2-10 days). PE was diagnosed in 1826 (53.0%) patients, while 1537 (45.0%) patients were diagnosed with DVT. Although most patients reported symptoms lasting <7 days, 444 patients (13.0%) reported a symptom duration exceeding 2 weeks.

**Table 1:** Baseline characteristics of the overall cohort.

Characteristic	Total (*N* = 3423)
Age (y)	67 (54-78)
Women	1612 (47)
BMI^a^ (kg/m^2^)	26.9 (24.1-30.4)
Previous VTE	333 (10)
Provoked VTE	1652 (48)
Cancer	595 (17)
Symptom duration^b^	4 (2-10)
Symptom duration (d)	
0-7	2477 (72)
8-14	502 (15)
Beyond 15	444 (13)
Diagnosis period	
2005-2009	688 (20)
2010-2014	1,205 (35)
2015-2020	1530 (45)
D-dimer (μg/mL)	3.6 (2.0-8.9)
VTE type	
PE	1826 (53)
DVT	1537 (45)
Proximal	1051 (68)
Distal	486 (32)
Axial	400 (82)
Muscle vein	86 (18)
UEDVT	60 (2)
Treatment	
LMWH	788 (25)
VKA	1098 (35)
DOAC	1519 (39)
Thromboloysis or UFH	8 (0.5)
None	10 (0.5)

Values are *n* (%) or median (IQR).

BMI, body mass index; DOAC, direct oral anticoagulant; DVT, deep vein thrombosis; LMWH, low-molecular-weight heparin; PE, pulmonary embolism; UEDVT, upper extremity DVT; UFH, unfractionated heparin; VTE, venous thromboembolism.

a Missing, 857

b Range, 0-365 days.

### 3.1 Correlation between D-dimer and symptom duration

**Figure 2:**
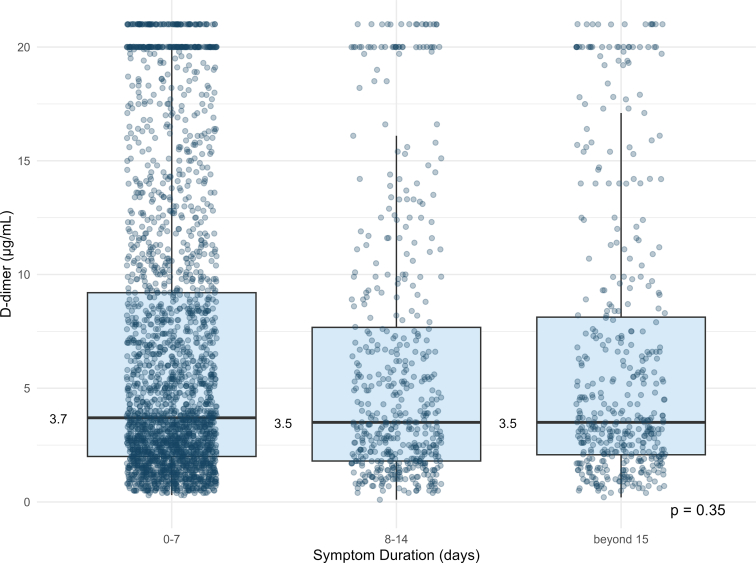
Median D-dimer values stratified by symptom duration. Values are presented across 3 categories: 0 to 7 days, 8 to 14 days, and beyond 15 days. Median D-dimer values are indicated to the left of each symptom duration category. Differences across categories were assessed using the Kruskal–Wallis test.

The median D-dimer were 3.7 μg/mL (IQR, 2.0-9.2 μg/mL) among patients with symptoms lasting 0 to 7 days, 3.5 μg/mL in those with symptoms lasting 8 to 14 days (IQR, 1.8-7.7 μg/mL), and 3.5 μg/mL (IQR, 2.1-8.1 μg/mL) in those with symptoms ≥15 days. No statistically significant differences were observed across the 3 categories (*P* = .35) (Figure 2).

**Figure 3:**
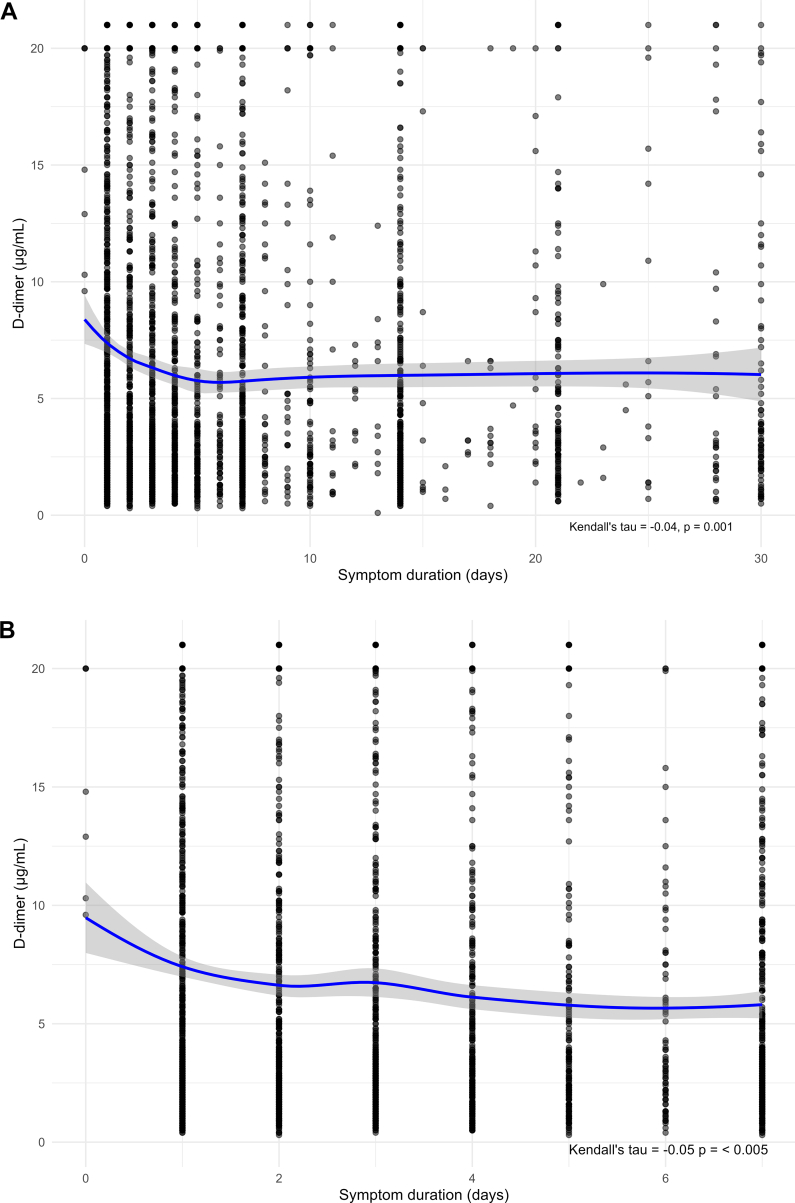
Association between D-dimer and symptom duration. (A) Scatter plot with locally estimated scatterplot smoothing (LOESS) smoothing, truncated at 30 days on the *x*-axis for improved visualization (*n* = 3423). (B) Subgroup analysis restricted to patients with symptom duration ≤7 days (*n* = 2477); 95% CI for the tau value in (A): −0.064 to −0.014; 95% CI for the tau value in (B): −0.080 to −0.023.

Figure 3 and Supplementary Figure 1 illustrate the association between D-dimer and symptom duration, together with results from the Kendall τ correlation test. Although the correlation was statistically significant (*P* < .005), the coefficient was very weak (−0.04; 95% CI, −0.064 to −0.014) (Figure 3A). As the visual inspection suggested a potentially stronger association during the first 7 days, an additional analysis was performed, restricted to patients with symptom duration ≤7 days (*n* = 2477). This analysis yielded a similar result (τ = −0.05; 95% CI, −0.08 to −0.023) (Figure 3B).

### 3.2 False-negative D-dimer results

Overall, 20 (0.6%) patients had a false-negative D-dimer at the conventional cutoff of 0.5 μg/mL, whereas 79 (2.3%) had false-negative D-dimer results when the age-adjusted cutoff was applied. The characteristics of both groups are summarized in Table 2. Most patients—18 of 20 (90.0%) at the conventional cutoff and 65 of 79 (82.2%) at the age-adjusted cutoff—were diagnosed between 2010 and 2020. In both groups, the majority were diagnosed with DVT (14/20 [70.0%] and 50/79 [63.0%], respectively). Among these, 50.0% of patients with false negatives according to the conventional cutoff and 66.0% according to the age-adjusted cutoff had distal DVTs. Notably, swelling was documented in 93.0% and 96.0%, respectively (Table 2).

**Table 2:** Characteristics and thrombus localization in patients with false-negative D-dimer results.

Characteristics	<0.5 (*n* = 20)	<Age-adjusted (*n* = 79)
Age (y)	49 (38-58)	68 (57-80)
Women	12 (60)	44 (56)
BMI (kg/m^2^)	27.4 (21.7-31.7)	26.7 (23.5-31.2)
Previous VTE	1 (9)	9 (21)
Unprovoked VTE	13 (65)	46 (58)
Cancer	3 (15)	11 (14)
Symptom duration	7 (2-14)	5 (2-14)
Symptom duration (d)		
0-7	13 (65)	49 (62)
8-14	4 (20)	16 (20)
Beyond 15	3 (15)	14 (18)
D-dimer (μg/L)	0.4 (0.35-0.40)	0.5 (0.45-0.60)
Diagnosis period		
2005-2009	2 (10)	14 (18)
2010-2014	7 (35)	31 (39)
2015-2020^a^	11 (55)	34 (43)
Diagnosis		
DVT	14 (70)	50 (63)
PE	3 (15)	25 (32)
UEDVT	3 (15)	4 (5)
DVT location		
Proximal	7/14 (50)	17/50 (34)
Distal	7/14 (50)	33/50 (66)
Axial	6/7 (86)	24/33 (73)
Muscle vein	1/7 (14)	9/33 (27)
DVT symptoms		
Swelling	13/14 (93)	48/50 (96)
Pain	13/14 (93)	44/50 (88)
Discoloration	2/14 (14)	13/50 (26)
PE symptoms		
Dyspnea	3/3 (100)	20/25 (80)
Pain	—	17/25 (68)

Patients are grouped by conventional cutoff (0.5 μg/mL, *n* = 20) and age-adjusted cutoff (*n* = 79). Values are *n* (%), *n*/*N* (%), or median (IQR).

BMI, body mass index; DOAC, direct oral anticoagulant; DVT, deep vein thrombosis; LMWH, low-molecular-weight heparin; PE, pulmonary embolism; UEDVT, upper extremity DVT; UFH, unfractionated heparin; VTE, venous thromboembolism.

a May 2020.

**Figure 4:**
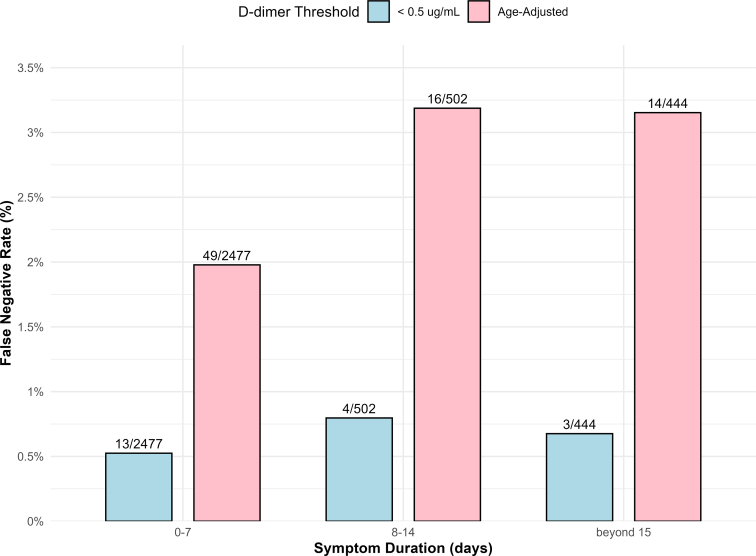
Proportion of patients with false-negative D-dimer results. Light blue bars indicate false-negative results at the conventional cutoff (0.5μg/mL), and pink bars indicated false-negative results using the age-adjusted cutoff. Values are stratified by symptom duration categories: 0 to 7 days, 8 to14 days, and beyond 15 days. False-negative D-dimer: Cochran–Armitage trend, *P* = .55. False-negative age-adjusted D-dimer: Cochran–Armitage trend, *P* = .06.

The proportion of patients with false-negative D-dimer results according to both conventional and age-adjusted cutoffs, stratified by symptom duration categories, is presented in Figure 4. The Cochran–Armitage trend test did not reveal statistically significant results for either group (*P* = .55 and *P* = .06, respectively) (Figure 4). Median D-dimer values and false-negative rates from subgroup analyses stratified by anticoagulant treatment, previous VTE, and VTE localization are presented in Supplementary Table 2.

### 3.3 Sensitivity analysis

Of the 3423 patients, 428 (12.5%) were diagnosed with DVT between January 2017 and May 2020. Among these, 307 (72.0%) patients reported symptoms within 7 days, 83 (19.0%) reported symptoms lasting 8 to 14 days, and 38 (9.0%) reported symptoms persisting ≥15 days. Supplementary Figure 2 shows a pattern of negative association between D-dimer and symptom duration in this subgroup similar to that observed in the overall cohort. Kendall τ correlation yielded a coefficient of −0.06 (*P* = .10), indicating a very weak correlation. Median D-dimer decreased from 3.0 μg/mL in patients with symptoms ≤7 days to 2.3 μg/mL in those with symptoms ≥15 days. However, the Kruskal–Wallis test did not demonstrate a statistically significant difference across the 3 categories (*P* = .58) (Supplementary Figures 3–5).

## 4 Discussion

In this study, we observed a negative correlation between D-dimer and symptom duration. However, the correlation was very weak, suggesting no clinical relevance despite statistical significance. Furthermore, stratifying D-dimer values according to symptom duration did not demonstrate statistically significant differences across the symptom duration categories. Additionally, analyses of patients with false-negative D-dimer results showed no association between symptom duration and false-negative results.

Traditionally, physicians have been advised to avoid D-dimer testing in patients with suspected VTE who report symptoms lasting longer than 1 to 2 weeks [8,9]. This recommendation is primarily based on studies published in 1990s, none of which specifically evaluated the correlation between D-dimer and symptom duration [6,7]. Moreover, one of the most frequently cited studies used the Vidas D-dimer assay (bioMérieux) [6], which is currently applied in <5% of laboratories worldwide [18]. More recent studies that attempted to examine the association between D-dimer and prolonged symptom duration, were small with few false-negative results [10,11].

While interpretation of correlation strength remains debated, most would consider coefficients >0.90 (positive or negative) to indicate strong associations, whereas values <0.10 suggest negligible to weak correlations [19]. In this study, we observed a negligible to weak negative correlation between D-dimer results and symptom duration. The discrepancy between the highly significant *P* value and the very low correlation coefficient potentially reflects the large sample size, which allowed detection of minimal trends. In this context, this may suggest that the statistically significant finding has limited clinical importance. This interpretation is supported by the lack of significant difference when D-dimer results were compared across symptom duration categories.

One previous study reported a slightly stronger, but still a weak negative correlation, than ours (Spearman ρ = −0.23; *P* = .001) [10]. However, that study included only 197 patients with confirmed VTE and provided no visual exploration of the data [10].

From a clinical perspective, the key question is whether prolonged symptoms increase the risk of false-negative D-dimer results. In this study, only 0.6% of patients had false-negative D-dimer results according to the conventional cutoff, consistent with findings from recent management studies that incorporated D-dimer testing regardless of the clinical probability and symptom duration [3,4]. Although the median symptom duration was longer in the false-negative group than that in the overall cohort (7 vs 4 days), the distribution of false-negative D-dimer results across the 3 symptom categories was comparable and showed no significant trend. Comparisons with prior studies are challenging, as most reported only a small number of false-negative results (*n* = 1-4) [7,11,20]. In contrast, substantially more false-negative results were observed when applying the age-adjusted cutoff. The proportion of false-negative results across symptom duration categories demonstrated a trend toward statistical significance, suggesting that the age-adjusted cutoff, at least for the STA-Lia-D Di assay, may be less reliable than the conventional cutoff in patients with prolonged symptoms.

The strength of this study is the large cohort of patients with objectively confirmed VTE diagnosis, including 946 (28%) who reported symptoms lasting >1 week. The study further provides detailed analyses, including stratification by symptom duration categories and characterization of false-negative results, which is particularly relevant given the skewed distribution of data. In addition, the use of a single D-dimer assay throughout the study period eliminates interassay variability.

### 4.1 Limitations

This study has several limitations. Only patients with available D-dimer results were included, which likely reflects a population with low to intermediate pretest probability. Consequently, the findings may not be generalizable to patients at high clinical risk for VTE. However, the sensitivity analysis, which included D-dimer testing all patients with suspected DVT regardless of pretest probability, produced similar results. In addition, 1248 patients (25%) were excluded due to unavailable D-dimer results or symptom duration data, which may have introduced selection bias. The range of symptom duration extended up to 1 year, which may seem implausible from a clinical perspective. To address this, all reported durations exceeding 3 months were crosschecked against the electronic medical records to confirm accuracy. Another limitation concerns interassay variability in D-dimer testing, which is well established [18,21]. Therefore, our findings may not be directly applicable to settings where assays other than those used in this study are used. Previous studies have indicated that anticoagulant treatment may reduce D-dimer sensitivity, potentially leading to false-negative results [22,23]. Although prior anticoagulant treatment was recorded, we were unable to distinguish between patients on long-term anticoagulant treatment and those who had received preemptive anticoagulation, which, furthermore, is not captured specifically in the TROLL registry. Therefore, we cannot exclude that some false-negative D-dimer results were attributable to pre-emptive anticoagulant treatment. Finally, our study design has inherent limitations regarding the identification of false-negative D-dimer results. We cannot exclude the possibility of false-negative D-dimer results among some of the 1248 patients who were ineligible due to missing D-dimer or symptom duration data. In addition, while the reason for proceeding with imaging despite negative D-dimer results likely reflected physicians overriding standard diagnostic procedures based on clinical judgment, the specific rationale for these decisions was not documented. Furthermore, patients with negative D-dimer results who did not develop VTE during follow-up were not captured in the registry, nor were patients with negative D-dimer results who developed VTE outside the registry’s catchment area. These factors may have led to an underestimation of false-negative D-dimer results.

## 5 Conclusion

The statistically significant association between D-dimer and symptom duration observed in this study is unlikely to be clinically meaningful, given the very low correlation coefficient. Importantly, the number of false-negative D-dimer results at the conventional cutoff of 0.5 μg/mL was small, and no association with symptom duration was identified. However, these findings warrant confirmation in a prospective study.
