# Supplementary material for: The impact of symptom duration on D-dimer: results from the Venous Thrombosis Registry in Østfold Hospital
Source: Res Pract Thromb Haemost. 2026 Mar 26;10(3):103457. doi: 10.1016/j.rpth.2026.103457 (PMC13156636; doi:10.1016/j.rpth.2026.103457)
Supplement: Supplementary Material [file mmc1.docx]

# Supplementary material

Supplementary figure S1. Scatter plot showing all patients, with symptom duration displayed across the full range.


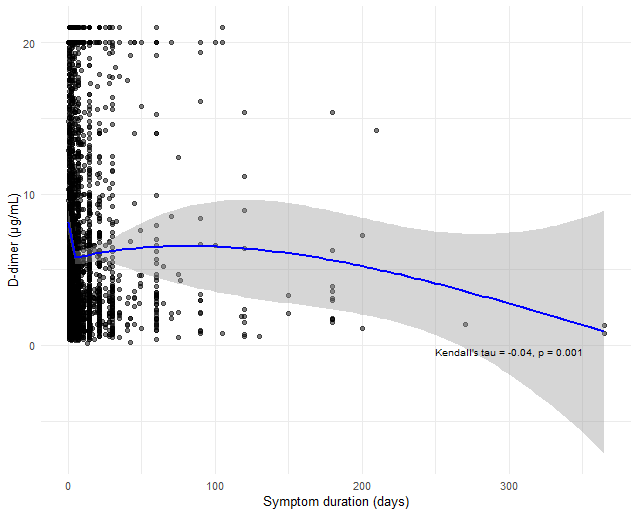


Supplementary Figure S2. Scatter plot displaying data restricted to patients diagnosed with DVT between 2017 and 2020 (n=428). The x-axis is truncated at 30 days for enhanced visualization.


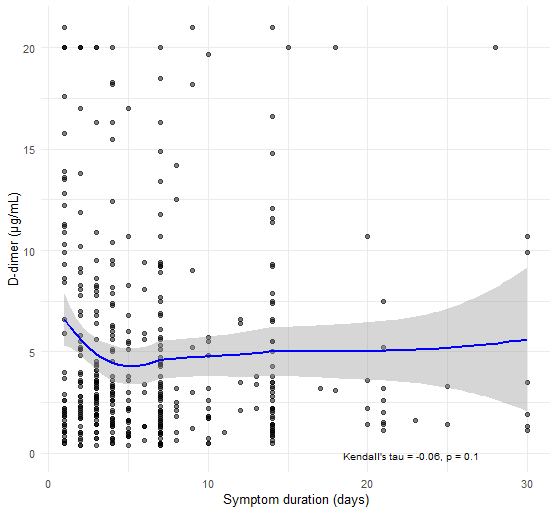


Supplementary Figure S3. Median D-dimer values stratified by symptom duration restricted to patients diagnosed with DVT between 2017 and 2020 (n=428). Values are presented across three categories: 0-7 days, 8-14 days, and beyond 15 days.


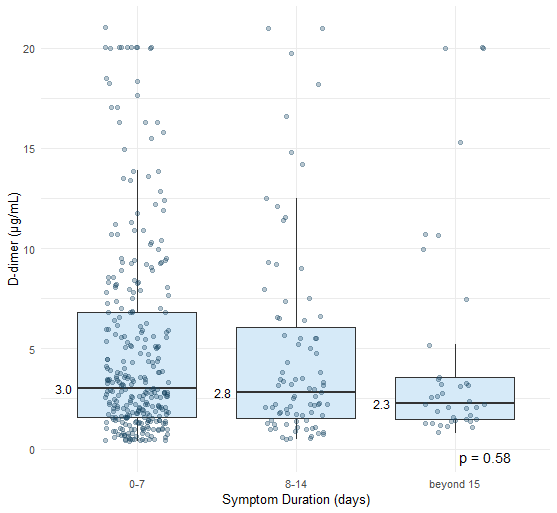


Median D-dimer values indicated to the left of each symptom duration category. Differences across categories were assessed using the Kruskal-Wallis test.

Supplementary Figure S4. Scatter plot displaying data restricted to patients diagnosed with proximal DVT between 2017 and 2020 (n=263). The x-axis is truncated at 30 days for enhanced visualization.


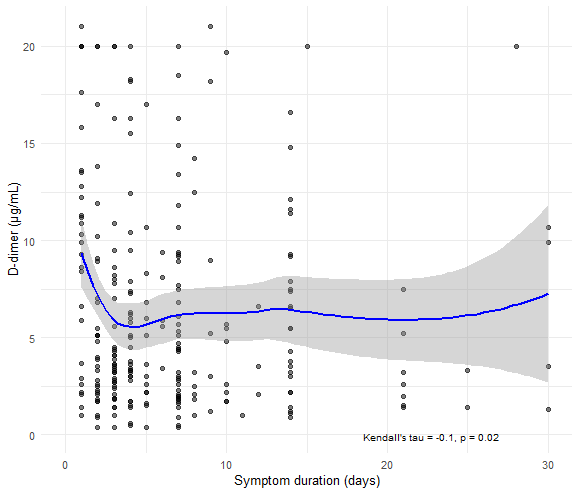


Supplementary Figure S5. Median D-dimer values stratified by symptom duration restricted to patients diagnosed with proximal DVT between 2017 and 2020 (n=263). Values are presented across three categories: 0-7 days, 8-14 days, and beyond 15 days.


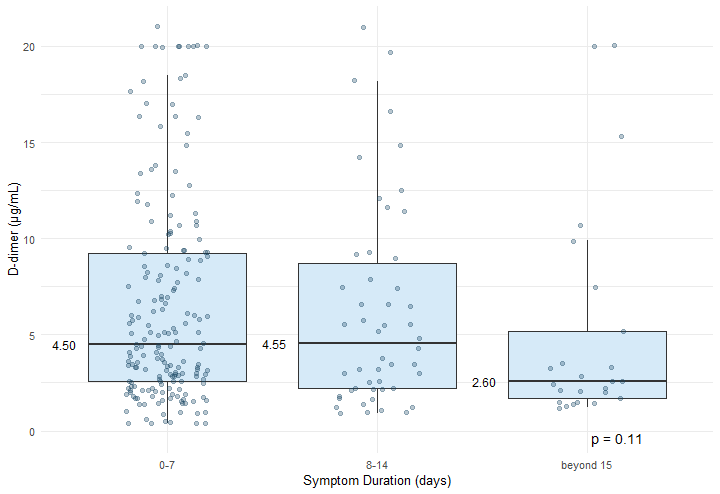


Supplementary Table S1. Baseline characteristics of the overall study cohort and of patients with DVT, PE, or UEDVT who were excluded due to unavailable D-dimer and/or symptom duration.

| Charachterstics | Total  N = 3,423 | Excluded  N = 1,111 |
| --- | --- | --- |
|  |  |  |
| Age, median (IQR) | 67 (54-78) | 68 (58-78) |
| Women, n (%) | 1,612 (47) | 561 (50) |
| BMI, median, (IQR) | 26.9 (24.1-30.4) | 25.7 (22.9-29.5) |
| Previous VTE, n (%) | 333 (10) | 77 (7) |
| Provoked VTE, n (%) | 1,652 (48) | 794 (71) |
| Cancer, n (%) | 595 (17) | 480 (43) |
|  |  |  |
| VTE type, n (%) |  |  |
| PE | 1,826 (53) | 726 (65) |
| DVT | 1,537 (45) | 356 (32) |
| UEDVT | 60 (2) | 29 (3) |
|  |  |  |
| Treatment, n (%) |  |  |
| LMWH | 788 (25) | 563 (50) |
| VKA | 1,098 (35) | 229 (21) |
| DOAC | 1,519 (39) | 269 (24) |
| Other/none | 18 (1) | 50 (5) |

Supplementary Table S2. Median (IQR) D-dimer values and false-negative rates stratified by prior anticoagulant treatment, previous VTE, and VTE localization. UEDVT patients (n=60) were excluded from the VTE localization analysis.

| Variable | Patients | D-dimer (µg/mL)  median (IQR) | p-value^*^ | D-dimer negative (<0.5µg/mL) | False-negative rate (95% CI) |
| --- | --- | --- | --- | --- | --- |
| Prior AC treatment |  |  |  |  |  |
| No | 3123 | 3.7 (2.0-7.3) | <0.001 | 16 | 0.51% (0.32-0.83) |
| Yes | 300 | 3.1 (1.8-5.8) |  | 4 | 1.33% (0.52-3.38)^+^ |
|  |  |  |  |  |  |
| Previous VTE |  |  | 0.07 |  |  |
| No | 3090 | 3.5 (1.9-8.0) |  | 19 | 0.61% (0.39-0.96) |
| Yes | 333 | 3.7 (2.1-9.3) |  | 1 | 0.30% (0.05-1.68)^++^ |
|  |  |  |  |  |  |
| VTE localization |  |  |  |  |  |
| PE | 1826 | 4.3 (2.2-10.6) | 0.86 | 3 | 0.16% (0.06-0.48) |
|  |  |  |  |  |  |
| DVT localization |  |  | <0.001 |  |  |
| Proximal | 1051 | - 1. (2.5-9.2) |  | 7 | 0.67% (0.32-1.37) ^+++^ |
| Distal | 486 | 1.7 (1.0-3.0) |  | 7 | 1.44% (0.70-2.94)^++++^ |

AC: anticoagulant

VTE: venous thromboembolism

PE: pulmonary embolism

DVT: deep vein thrombosis

^*^: Mann-Whitney U test

+: Odds ratio: 2.62 (95% CI, 0.63-8.20) for a false-negative D-dimer result in the prior AC group compared to the no prior AC group.

++: Odds ratio: 0.49 (95% CI, 0.01-3.08) for a false-negative D-dimer result in the previous VTE group compared to the no previous VTE group.

+++: Odds ratio: 4.07 (95% CI, 0.93-24.46) for a false-negative D-dimer result in the proximal DVT group compared to the PE group.

++++: Odds ratio: 2.18 (95% CI, 0.65-7.32) for a false-negative D-dimer result in the distal DVT group compared to the proximal DVT group.
